# The periplasmic binding protein NrtT affects xantham gum production and pathogenesis in *Xanthomonas citri*

**DOI:** 10.1002/2211-5463.12281

**Published:** 2017-09-13

**Authors:** Aline Sampaio, Vanessa Rodrigues Pegos, Elisa Emiko Oshiro, Andrea Balan

**Affiliations:** ^1^ Programa Interunidades em Biotecnologia Instituto de Ciências Biomédicas Universidade de São Paulo USP Brazil; ^2^ Laboratório Nacional de Biociências (LNBio) Centro de Pesquisas em Energia e Materiais (CNPEM) São Paulo Brazil; ^3^ Post‐Graduate Program in Genetics and Molecular Biology Institute of Biology University of Campinas UNICAMP Campinas São Paulo Brazil; ^4^ Departmento de Microbiologia Instituto de Ciências Biomédicas Universidade de São Paulo Brazil

**Keywords:** ABC transporter, alkanesulfonates/taurine, NrtT, pathogenesis, periplasmic binding protein, *Xanthomonas citri*

## Abstract

In *Xanthomonas citri*, the bacterium that causes citrus canker, three ATP‐binding cassette (ABC) transporters are known to be dedicated to the uptake of sulfur compounds. In this work, using functional, biophysical and structural methods, we showed that NrtT, a periplasmic component of the ABC transporter NrtCB, is an alkanesulfonate‐binding protein and that the deletion of the *nrtT* gene affected xantham gum synthesis, adhesion and biofilm production, similarly to the phenotype obtained in the *X. citri ssuA*‐knockout strain, in which the alkanesulfonate‐binding protein SsuA is absent. Although NrtA and SsuA share similar ligands, the function of these proteins is not complementary. These results emphasize that organic‐sulfur sources are directly involved with bacterial infection *in vivo* and are needed for pathogenesis in *X. citri*.

**Abbreviations**
CFU: colony forming unit
DLS: dynamic light scattering
KEGG: Kyoto Encyclopedia of Genes and Genomes
PDB: Protein Data Bank
SAXS: small angle X‐ray scattering

*Xanthomonas citri* is a gram‐negative phytopathogenic bacterium that causes one of the most devastating diseases, citrus canker, affecting many important citrus varieties 1, 2 including *Citrus sinensis* (or sweet orange). The citrus canker is manifested by the accumulation of water and formation of chlorosis rings, necrotic lesions of brown color often surrounded by a yellow halo in leaves and fruits, which reduce quality and yield 3, 4, 5. As reported by the US Department of Agriculture Foreign Agricultural Service, Brazil has been for many years the largest producer and exporter of orange juice, and such damage hampers commercialization and export, reducing the economy and the generation of jobs in the country 6, 7. Although many chemical methods have significantly reduced the incidence of canker lesions on fruit when compared with the untreated control 8, as yet only palliative measures are used to contain citrus canker. The study and comprehension of mechanisms that the bacterium uses to infect, grow and induce pathogenesis is important for the development of new strategies of control. Characterization of components of the cellular envelope and membrane proteins involved in transport may lead to their use as targets for antimicrobial therapy and agricultural pesticides 9.

ATP‐binding cassette (ABC) transporters are integral membrane proteins whose fundamental role is the translocation of a variety of substrates across the cell membrane in a process dependent on ATP hydrolysis 10. They can be classified as importers or exporters depending on the direction of transport, always against the concentration gradient 11. Importers typically function in the uptake of nutrients such as peptides, ions and sugars, and they play an important role in metabolism and pathogenesis, including in *X. citri*. According to the Kyoto Encyclopedia of Genes and Genomes (KEGG) database, *X. citri* has at least 87 genes encoding components of ABC transporter‐type importers, whose importance has been shown *in vitro* and *in vivo*, including in the attenuation and delay of canker symptoms, loss of pathogenicity and reduction of cell density when compared with the wild strain in infection tests 12, 13, 14, 15, 16. Furthermore, from the list of importers, 14 genes are related to the uptake of nitrate/taurine or sulfate/aliphatic sulfonates and akanesulfonates, showing that these compounds might confer on the bacterium some advantage during its growth and evidencing the relevance of sulfur in this process. In fact, during growth of the bacterium in LB broth and XAM1 media, the ABC transporters responsible for sulfate uptake (SbpCysUWA) and the additional systems related to the transport of alkanesulfonates and/or organosulfur compounds (SsuABC1 and SsuABC2) are differentially induced 17, suggesting a regulation based on sulfur availability. Studies have shown a significant and continuous activation of the *sbpcysUWA* promoter during the first stages of infection of *X. citri* in *C. sinensis* leaves, indicating the need for sulfate in these conditions 18. Moreover, the deletion of the *X. citri ssuA2* gene, which encodes an alkanesulfonate‐binding protein, SsuA, drastically affected xanthan gum production *in vitro* and the development of canker lesions when the bacterium infected *C. sinensis* leaves 14. Although there is no direct relationship between sulfur compounds and xantham gum, the depletion of these molecules directly affects the synthesis of amino acids such as cysteine and methionine with general consequences for metabolism and protein synthesis. In fact, the amino acid sequences of the 12 proteins involved in the xantham gum pathway (GumB–GumM) have at least four cysteines each 19. With all this evidence it is worth better understanding the relationship between ABC transporters dedicated to uptake of sulfur containing molecules and *X. citri* infection and pathogenesis.

In the present work, we investigated the role of the periplasmic component (NrtT) of the single operon‐like gene cluster (*tauDnrtTCDB*) putatively involved in nitrate/sulfonate/taurine assimilation. Bioinformatics analyses and biophysical and functional characterization of the periplasmic binding protein NrtT were performed in order to identify its ligand and its role in the bacterium's physiology. We showed that the sulfonates MOPS and taurine induced structural changes and an increase in the thermal stability of NrtT, and that the protein is necessary for *in vivo* growth when these compounds are the unique source of sulfur, strongly suggesting a role for NrtT in the binding of aliphatic sulfonates/alkanesulfonates. Finally, the *nrtT‐*knockout mutant of the *X. citri* strain showed a reduction in xanthan gum production, adhesion and development of canker lesions in *C. sinensis*. Altogether, these results are in accordance with the previous literature and emphasize the relevance of ABC transporters dedicated to the uptake of sulfur compounds in the pathogenesis of *X. citri*.

## Experimental procedures

### Characterization of the operon *tauDnrtTCDB* and bioinformatics analysis

The genome organization of operons from different species and primary sequence of the proteins were obtained from the KEGG database (http://www.genome.jp/kegg/). A search for proteins with sequence similarity was made with the blastp program using non‐redundant search and similar proteins with resolved structure were found using blastp against the Protein Data Bank (PDB) 20. Amino acid sequence alignment was performed using clustal omega 21. The three‐dimensional model of NrtT was built using the modeller program 22 based on the structural coordinates of the *X. citri* alkanesulfonate‐binding protein SsuA2 (PDB code 3E4R). Model validation was performed using tools from the wincoot program 23. Figures were drawn using the pymol program 24.

### Cloning of *nrtT* gene, expression and purification of NrtT

The nucleotide sequence of the gene encoding the NrtT protein was obtained from the KEGG database and was analyzed by the structural biology helper prediction program (http://lge.ibi.unicamp.br/lnbio/index2.php?refer=2), in which we identified an 84 bp region corresponding to the signal peptide. The *nrtT* gene was amplified according to standard protocols 25, cloned into the pGEM T‐Easy vector (Promega, Madision, WI, USA) and subcloned into the pET28a‐SUMO for expression. All DNA from clones was sequenced. Cultures of the recombinant *Escherichia coli* BL21 (DE3) strain transformed with pET28a.sumo.nrtT were prepared aerobically in LB broth supplemented with 50 μg·mL^−1^ kanamycin until mid‐log phase (*D*_600_: 0.5–0.6); IPTG was then added to a final concentration of 0.45 mm and the cultures were induced aerobically (250 r.p.m.) for 4 h at 30 °C. Cells were collected by centrifugation at 8000 ***g*** for 15 min at 4 °C and stored at −20 °C for approximately 16 h before lysis. The cell pellets were suspended in buffer I (50 mm sodium phosphate buffer, pH 7.4, containing 150 mm NaCl, 5% glycerol and 1 mm phenylmethylsulfonyl fluoride) and incubated with lysozyme (final concentration of 250 μg·mL^−1^) for 1 h in an ice bath. The cells were lysed in a disrupter (LA Biosystems BV, Waalwijk, the Netherlands) and centrifuged at 16 000 ***g*** for 45 min at 4 °C. The filtered supernatant was first purified by immobilized metal affinity chromatography using the HisTrap HP 5 mL (GE Healthcare, San Diego, CA, USA) column pre‐equilibrated in buffer I. Elution fractions were obtained in two steps with buffer I with added 500 mm imidazole and concentrated with an Amicon ultra filter (Millipore, Billerica, MA, USA; molecular mass cut‐off 10 kDa). The recombinant SUMO‐NrtT protein was cleaved with 1 mg of SUMO protease to each 5 mg of protein for 16 h at 4 °C. The cleaved sample was subjected to molecular size exclusion chromatography with a HiLoad Superdex 75 16/60 column (GE Healthcare), using buffer I. Separation of the cleavage products were confirmed by SDS/PAGE using 15% acrylamide gels. Protein concentration was determined spectrophotometrically using a Nanodrop spectrophotometer (Thermo Fisher Scientific, Waltham, MA, USA) from the values of the molar extinction coefficient and the absorbance at 280 nm.

### Biophysical experiments

Samples of purified NrtT (80 μL, 1 mg·mL^−1^) were previously centrifuged at 12 000 ***g*** for 30 min and analyzed by dynamic light scattering with DynaPro 99 equipment (Wyatt Technology, Santa Barbara, CA, USA) at 18 °C. For the analysis, 300 acquisitions of 10 s each were collected. For circular dichroism measurements, protein and ligands were diluted in Milli‐Q water to a final concentration of 3 and 50 μm, respectively. For each spectrum, 20 accumulations were collected at 50 nm·min^−1^ at 10 °C. Each thermal denaturation was monitored from 10 to 100 °C with 1 °C·min^−1^ intervals. Both experiments were performed on a Jasco J‐180 spectro‐polarimeter (Jasco, Easton, MD, USA) equipped with a Peltier device for temperature control. Fluorescence intrinsic to tryptophan was evaluated for NrtT in the absence (3 μm protein) and presence of 10 μm MOPS and taurine. The excitation wavelength was 280 nm and data acquisition was obtained between 300 and 400 nm at 1 nm·s^−1^ in a PC1/K2 spectrofluorimeter (ISS, Champaign, IL, USA).

### Small angle X‐ray scattering analysis

NrtT small angle X‐ray scattering (SAXS) analyses in the absence and presence of MOPS were carried out on the SAXS1 beamline of the Brazilian National Laboratory of Synchrotron Light (LNLS; Campinas, SP, Brazil). Measurements of buffers and samples were made at room temperature with exposure time of 300 s. Samples were prepared with 10 μm of NrtT and 30 μm of MOPS. Subtraction of buffers spectra was obtained with the fit‐2d program 26. Envelopes were determined from *ab initio* molecular modeling using the atsas 2.1 software package 27 and the graphs were constructed in the program originpro 8 (OriginLab Corp., Northampton, MA, USA). The rigid body model was created from the coral program, included in the atsas 2.1 package, and the generated structure was observed in the pymol program.

### Construction of the *Xac::nrtT* mutant and the complementary strain *Xac::nrtTc*

Site‐specific inactivation of the *nrtT* gene was conducted in a strain of *X. citri* 306 using a suicide plasmid according to previously published procedures 12 (Fig. 6A,B). Construction of the vector pKX33.pnrtT used for complementation of *Xac::nrtT* was commercially synthesized by GenOne Biotechnologies (Rio de Janeiro, RJ, Brazil). For that, the full *nrtT* gene was cloned immediately downstream of the 1000 bp sequence upstream of the *nrtBCDT* operon that included the promoter region previously cloned into the pKX33 plasmid 28. Two pairs of oligonucleotides containing sites for cleavage with restriction enzymes were designed to screen and monitor the complementary gene. For the amplification of the promoter region and *nrtT* gene, respectively, we used the following set of oligonucleotides: (a) forward 5′‐GTCGACCGTTGCTGGATATCGACGC‐3′ and reverse 5′‐GAATTCGGGGGAAGACGCATCGC‐3′, and (b) forward 5′‐CCCACGAATTCTTGCCGCACC‐3′ and reverse 5′‐CGAGGATCCTCAGCTCCCGC‐3′. The vector pKX33.pnrtT was used to transform the *Xac::nrtT* competent strain by electroporation according Oshiro and collaborators 12. The selection of the colonies containing the complementary gene was made from the growth of the culture transformed in LB broth with ampicillin, kanamycin and spectinomycin followed by analysis of colony PCR amplification.

### Quantification of xanthan gum production

Analysis of xanthan gum production was carried out according to the protocol previously published 29. The *Xac* and *Xac::nrtT* strains were grown in LB broth with the appropriate antibiotics at 28 °C for 16 h with shaking at 250 r.p.m. After growth, cultures of the mutant and the wild strains were diluted in sterile Milli‐Q water until absorbance at 600 nm of 0.1 [10^6^ colony forming unit (CFU)·mL^−1^] and inoculated into 100 mL of LB broth with ampicillin. Growth was maintained for 24 h and the absorbance at 600 nm was measured. Samples were centrifuged at 13 780 ***g***, 4 °C for 5 min. The pellets were resuspended in 100 mL of 2% potassium chloride and 200 mL of ethanol and then centrifuged at 8200 ***g***, 4 °C for 20 min. The supernatants were transferred to pre‐weighed Falcon‐type conical tubes and subjected to lyophilization for 18 h. The lyophilized gum was weighed for mass comparison between wild and mutant strains. Three independent samples were used for each strain.

### Analysis of cell adhesion and biofilm production

To measure the level of cells adhered to an abiotic surface, cultures of wild‐type, mutant and complementary strains were grown for 16 h in LB broth, at 250 r.p.m. at 28 °C followed by centrifugation at 20 000 ***g***. The cell pellets were resuspended in 10 mm Tris/HCl buffer pH 8.0 and cultures were normalized for 10^8^ CFU·mL^−1^; from these, aliquots of 200 μL were transferred to 1.5 mL sterile microtubes and incubated for 6 h at 28 °C. Adhesion was monitored by staining with 1% crystal violet for 45 min. The excess of dye was removed by washing with milli‐Q water with the aid of a pipette, and the crystal violet was solubilized by the addition of 250 μL of 99% ethanol in each tube. The amount of retained crystal violet was quantified in a UV‐visible spectrophotometer at 590 nm. The assay was repeated three times with 10 replicates at a time. Values are expressed as the means ± standard deviation after statistical treatment using Tukey's test.

To observe bacterial adhesion on biotic surface, cultures of 16 h were diluted to 10^4^ CFU·mL^−1^ in LB broth and 20 μL of the bacterial suspension were dripped at three distinct points on the leaves’ abaxial surface. The leaves were maintained at 30 °C for 24 h and adhesion was quantified by measuring the optical density of the recovered cells after dissolution in ethanol/acetone (70: 30, v/v). For visualization the leaves also were stained with 1% crystal violet and three steps of washing with distilled water to remove the sample excess on the leaf surface. The leaves were dried at room temperature and photographed. Statistical analysis was performed using Student's *t* test and *P*‐values < 0.05 were considered significant.

### *In vitro* and *in vivo* growth of the strains

*In vitro* growth of the samples was monitored after inoculation of 10^6^ CFU of *X. citri* and *Xac::nrtT* cultures into 50 mL (1 : 100) of LB broth, XAM1 and XAM1 modified media (XAM1a and XAM1b), where XAM1 is 3.0 mm (NH_4_)SO_4_, 16.5 mm KH_2_PO_4_, 30 mm K_2_HPO_4_, 0.85 mm Na_3_C_6_H_5_O_7_, 100 mm MgSO_4_, 0.18% (m/v) fructose, 0.34% (m/v) sucrose, 0.03% (m/v) casamino acids and 0.1% (m/v) BSA 30; in XAM1a 3.0 mm MOPS and 100 mm MgCl_2_ replace (NH_4_)SO_4_ and MgSO_4_, respectively; and in XAM1b 3.0 mm taurine replaces (NH_4_)SO_4_. Growth was maintained at 30 °C, 250 r.p.m. for 72 h. The absorbance at 600 nm was measured every 24 h. The experiment was carried out three times and results are represented by the mean and standard deviation.

To evaluate the growth of cells *in vivo*, cells were grown in glass Petri plates at 30 °C for 48 h. The cell mass on the surface was recovered and diluted in water to obtain an absorbance of 0.1 at 600 nm for *Xac* and *Xac::nrtTc* and 0.15 for *Xac::nrtT*, which was equivalent to 9 × 10^6^ viable cells in the inoculum. The dilutions were used to inoculate leaves of *C. sinensis* (sweet orange) as previously described 14. Plants of *C. sinensis* were obtained from certified nurseries and kept in a growth room at 25–28 °C with fluorescent light illumination. The appearance of canker pustules and lesion phenotypes was monitored daily for 12 days. To follow the growth of the bacteria inside the plant tissue, leaves were infiltrated with 7 × 10^6^ viable cells in three different sectors. Every other day, three circular sections 1 cm in diameter containing the bacterial infiltrates were macerated in water, and diluted aliquots were plated on LB broth agar plates for determination of the number of viable bacteria.

## Results

### Genomic organization of the putative ABC transporter *tauDnrtTCDB* of *X. citri* and comparison with transporters of aliphatic compounds

**Figure 1:**
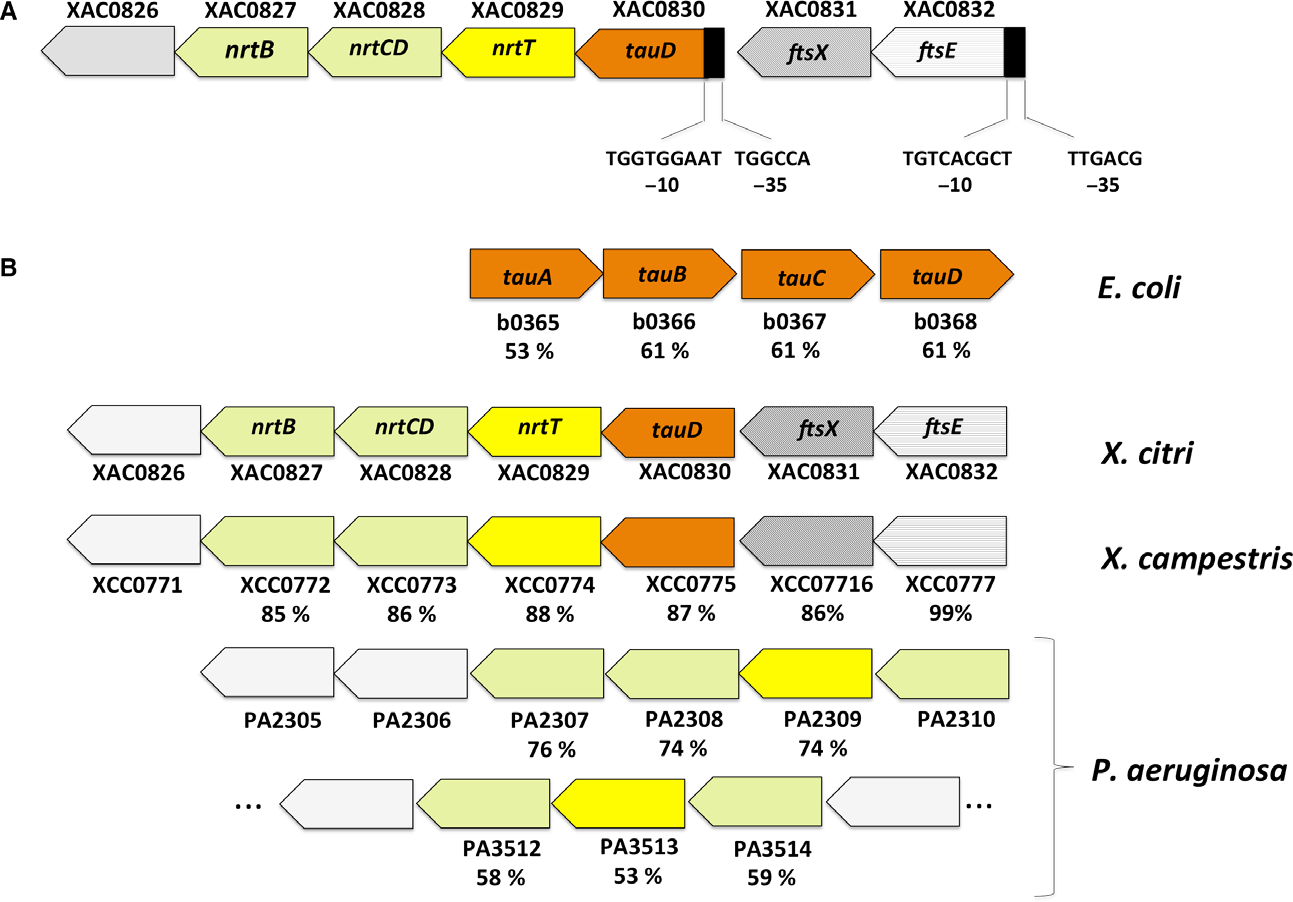
Genetic organization of gene *nrt* in *Xanthomonas citri* in comparison with *Xanthomonas campestris*,* Pseudomonas aeruginosa* and *Escherichia coli*. (A) Promoter regions (black boxes) predicted in the cluster of *X. citri* by the softberry bprom program. (B) Genetic organization of operons putatively involved with the transport and metabolism of mineral and organic ions and nitrate/nitrite/taurine. Orthologues and genes belonging to the same operon are represented in same colors, using the KEGG code and name. The percentage amino acid sequence identity is shown for each protein in comparison with *X. citri* orthologues.

In the *X. citri* genome the gene *nrtT* (Xac0829) is located dowstream of *tauD* (Xac0830) and upstream of genes *nrtCD* (Xac0828) and *nrtB* (Xac0827). According to the program softberry bprom (http://www.softberry.com), there is a promoter region upstream of *tauD* indicating that this gene also belongs to the same operon (Fig. 1A). Altogether, the genes encode a putative ABC transporter consisting of the periplasmic binding protein NrtT, two ATPases NrtCD and a permease NrtB (shown respectively as yellow and pale yellow) putatively related to the transport of mineral and organic ions (NitT/TauT family transporter). Corroborating the function predicted for the operon, *tauD* encodes a taurine dehydrogenase (orange), whose orthologue in *E. coli* is responsible for oxide reduction of taurine after its uptake from the cognate transporter TauABC (Fig. 1B). Upstream of the *X. citri tauDnrtTCDB* operon, genes *ftsE* and *ftsX* respectively encode the ATPase and membrane components of an ABC transport system involved in the process of cell division and salt transport in *E. coli* 31. The *ftsE* and *ftsX* genes of *X. citri* share 62% and 72% sequence identity with their orthologues in *E. coli*.

The comparison of NrtT with putative orthologues using the blastp tool confirmed similarities with nitrate‐binding proteins from different species of the genus *Xanthomonas* and the two alkanesulfonate‐binding proteins from *X. citri*, SsuA1 and SsuA2 17. Moreover, the same genome organization found in *X. citri* is present in *Xanthomonas campestris*, another important phytopathogen that infects citrus plants but with a different mechanism (Fig. 1B). Furthermore, NrtT also shares amino acid sequence identity of 53% and 74% with putative orthologues of *Pseudomonas aeruginosa* that are present in operons related to taurine transport (PA2309 and PA2513, respectively) and 53% with the taurine‐binding protein TauA of *E. coli* (Fig. 1B). In *P. aeruginosa* putative orthologues of NrtT belong to distinct loci: the first one (consisting of genes PA2305 up to PA2310) conserves part of the genome organization found in *X. citri* and also a gene encoding a taurine dehydrogenase (PA2310), which is implicated in l‐2‐amino‐4‐methoxy‐*trans*‐3‐butenoic acid production 32. The second operon (genes PA3512 to PA3514) encodes a putative ABC transporter and belongs to an extensive locus that represents a set of hypothetical proteins involved with metabolism of aldehydes, adenyl succinate and acetolactate and with cell division.

### NrtT periplasmic protein preserves essential residues for interaction with alkanesulfonates and aliphatic sulfonates

**Figure 2:**
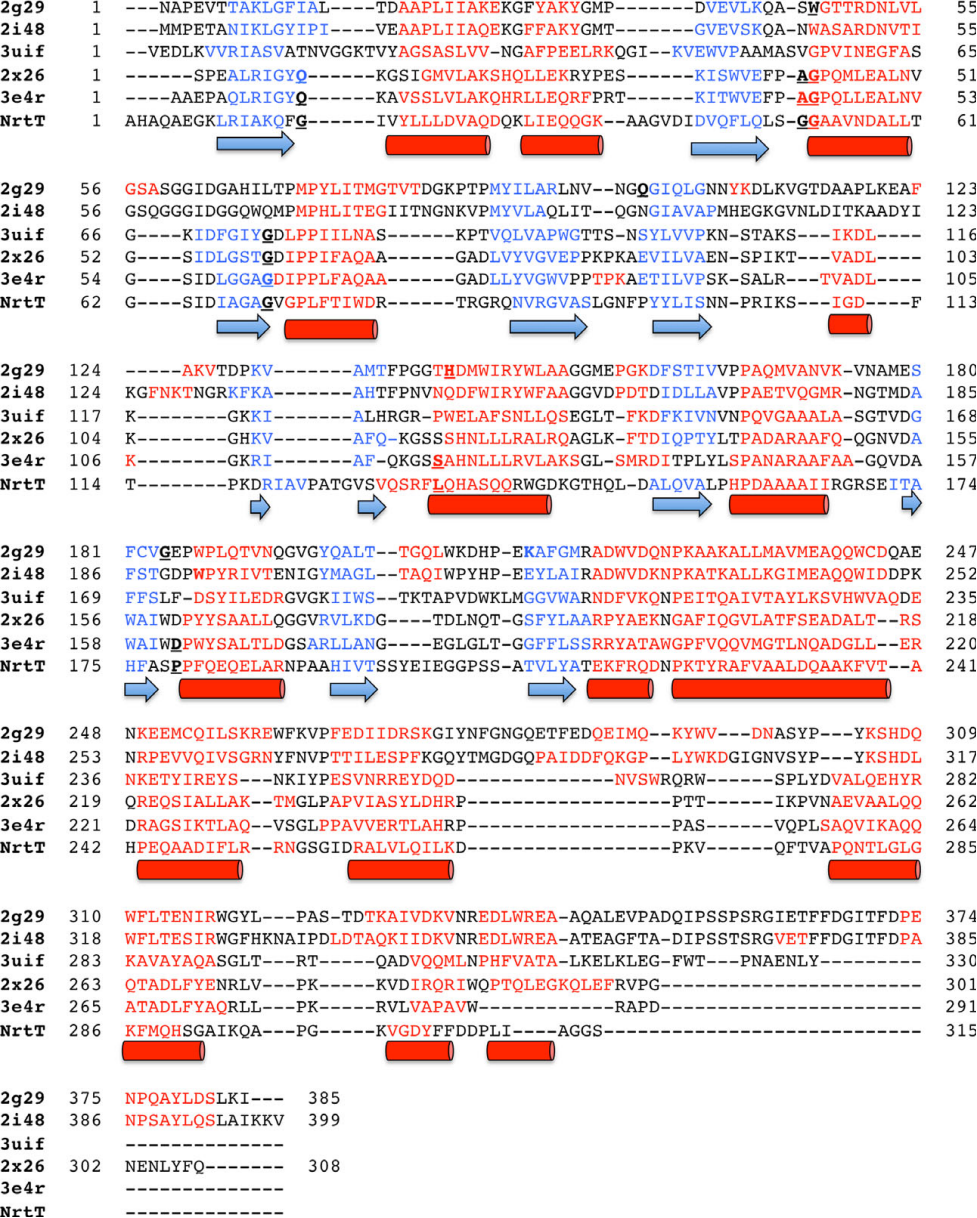
Multiple sequence and structure alignment of *Xanthomonas citri* NrtT model with proteins identified in the blastp against PDB. The alignment was performed by the promals3d program 48. The α‐helices and β‐sheets are colored in red (arrow) and blue (cylinders), respectively. Residues that interact with the ligands in the PDB structures are showed in bold underlined. The same was indicated for NrtT. The first three letter code refers to the PDB data bank. 2G29, nitrate‐binding protein NrtA from *Synechocystis* sp. PCC 6803; 2I48, bicarbonate‐binding protein CmpA from *Synechocystis* sp. PCC 6803; 2X26, sulfonate‐binding protein SsuA from *Escherichia coli*; 3E4R, sulfonate‐binding protein SsuA from *X. citri*; 3UIF, sulfonate‐binding protein SsuA from *Methylobacillus flagelatus* KT; NrtT, nitrate/alkanesulfonate/taurine‐binding protein NrtT from *X. citri*.

**Figure 3:**
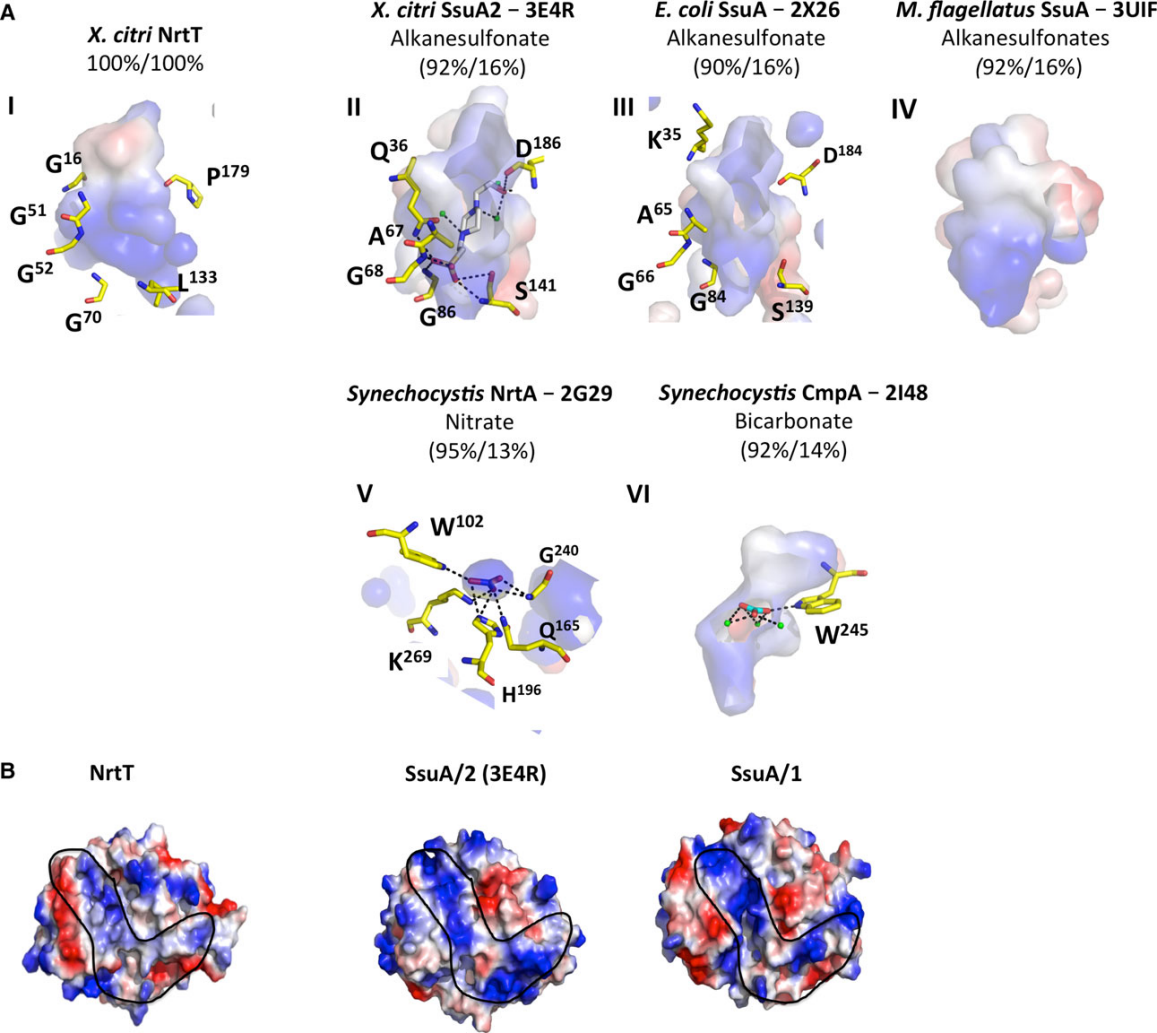
Comparison of the ligand‐binding pockets of NrtT and other proteins. (A) Surface electrostatic potential and residues from the ligand‐binding pockets of the five proteins identified after NrtT blast against the PDB evidencing that NrtT is closely related to the alkanesulfonate‐binding proteins. Charges are shown in red (negative) and blue (positive) and residues that interact with the ligands in the structures are shown as yellow sticks. Residues from NrtT pocket were predicted based on the structural alignment with the sulfonate‐binding proteins. Proteins name are showed with the PDB code and the ligands (except for NrtT and *Xanthomonas citri* SsuA/1). The percentages correspond to the query coverage and the amino acid sequence identity after alignment. 2G29, nitrate‐binding protein NrtA from *Synechocystis* sp. PCC 6803 bound to nitrate (blue stick); 2I48, bicarbonate‐binding protein CmpA from *Synechocystis* sp. PCC 6803 bound to bicarbonate (green stick); 2X26, alkanesulfonate‐binding protein SsuA from *Escherichia coli*; 3E4R, alkanesulfonate‐binding protein SsuA/2 from *X. citri* bound to HEPES (gray sticks); 3UIF, sulfonate‐binding protein SsuA from *Methylobacillus flagelatus* KT; NrtT, nitrate/alkanesulfonate/taurine‐binding protein NrtT from *X. citri*. (B) Comparison of the electrostatic potential at the pocket entrance of NrtT with the *X. citri* alkanesulfonate‐binding proteins SsuA/1 (Xac0849, model) and SsuA/2 (Xac3198, PDB code 3E4R) The proteins are shown as external surface and the black line shows the conserved profile of charges.

A search of protein structures related to the NrtT amino acid sequence in the PDB corroborated the previous results, revealing five structures of the following periplasmic binding proteins: nitrate‐binding protein NrtA and carbonate‐binding protein CmpA from *Synechocystis* PCC 6803 22, 33, and alkanesulfonate‐binding protein SsuA from *E. coli* 34 and *X. citri* 14, which shared up to 16% amino acid sequence identity. According to the structural alignment, all the proteins conserve the secondary structures but NrtT is closely related to the structures of alkanesulfonate‐binding proteins from *Methylobacillus flagelatus* (PDB 3UIF), *E. coli* (PDB 2X26) and *X. citri* (PDB 3E4R) 14 (Fig. 2). To compare the similarities in the ligand‐binding pocket of the proteins, we built a model of NrtT using the modeller program 28 based on the structural coordinates of *X. citri* SsuA, and performed a structural superimposition of all the proteins (Fig. 3). Analysis of the residues from the pocket and electrostatical potential showed that NrtT was grouped with the alkanesulfonate‐binding proteins SsuA from *X. citri*, SsuA from *E. coli* (PDB 2X26) and SsuA from *M. flagelatus* (PDB 3UIF; Fig. 3AI–IV). However, from these, the similarities between the pocket volumes, electrostatic potential and residues that interact with the ligands are evident only for the structures of SsuA from *X. citri* and *E. coli*. The ligand pockets in these two structures have very similar electrostatic potential that assumes a positive charge (blue color; Fig. 3AII,III). On the other hand, the NrtT pocket conserves the positive electrostatic potential at the bottom but not at the top, which is more negative (red color) and reveals the possibility of a different type of ligand (Fig. 3AI). In that sense, NrtT is closer to *M. flagelatus* SsuA, which unfortunately was solved in the apo conformation. Moreover, the data show that the *X. citri* NrtT pocket does not conserve structure or residues when compared with the structures of the nitrate‐binding protein NrtA or bicarbonate‐binding protein CmpA (Fig. 3AI,V,VI), excluding these molecules from the list of possible NrtT ligands.

Following the putative function of the alkanesulfonate‐binding protein for NrtT, we also compared the surface electrostatic potential of the entrance of the pocket in NrtT and the two paralogues (SsuA1 and SsuA2) of *X. citri* (Fig. 3B). Again, the results evidenced that despite some similarities in the general charges, the NrtT surface in this region shows significant differences compared with the two other proteins. In fact, the three proteins showed significant differences (Fig. 3B, black line contour).

### Circular dichroism and intrinsic fluorescence of tryptophans suggest that NrtT binds MOPS and taurine

**Figure 4:**
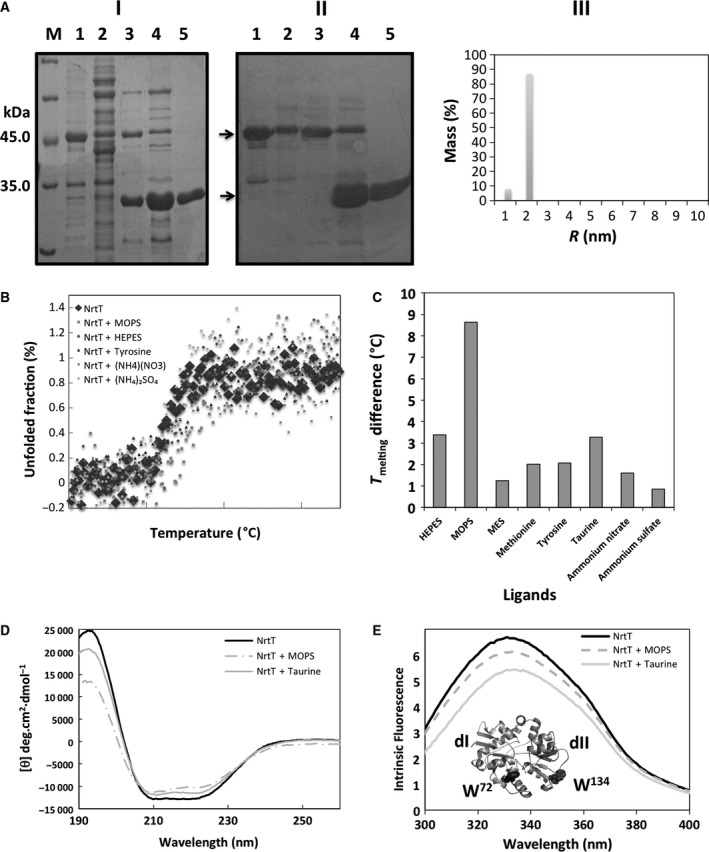
Production of *Xanthomonas citri* NrtT and biophysical analyses in the presence of putative ligands. (A) Production of NrtT from *Escherichia coli* BL21(DE3) cells. (I) 15% SDS/PAGE of NrtT samples obtained in different steps of expression, purification and cleavage with SUMO protease. (II) Western blot using antibodies against NrtT. M, molecular mass marker; 1, insoluble fraction of the *E. coli* BL21 (DE3) induced cells; 2, soluble fraction; 3, size‐exclusion chromatography sample of NrtT before cleavage with SUMO‐protease; 4, sample after cleavage with SUMO‐protease; 5, concentrated sample used for biophysical and structural assays. The arrows indicate the estimated molecular mass for SUMO‐NrtT (NC) and the estimated molecular mass of the NrtT after cleavage with SUMO‐protease (CL). (III) DLS plot showing that after SUMO protease cleavage NrtT was 89% monodisperse. (B) Thermal denaturation curve of NrtT in the presence of different molecules evidencing a gain of thermal stability in the presence of MOPS. (C) Plots of the different melting temperatures identified after thermal denaturation of NrtT in the absence and presence of different putative ligands. (D) Circular dichroism spectra of NrtT in the absence and presence of MOPS and taurine showing the α–β profile expected for periplasmic binding proteins and slight structural changes. (E) Intrinsic fluorescence of tryptophans from NrtT in the absence and presence of MOPS and taurine. The inset shows the NrtT structural model in cartoon with the two tryptophans (W^72^ and W^134^) shown as gray spheres. DLS, dynamic light scattering.

The *X. citri* NrtT was expressed as a soluble and monodisperse cytosolic protein genetically fused to the His_6_tag‐SUMO protein. After purification by immobilized metal affinity chromatography followed by size‐exclusion chromatography, the recombinant protein was cleaved with thrombin showing a molecular mass of 34 kDa (Fig. 4A). The analysis of thermal denaturation of NrtT in the absence and presence of different molecules, including alkanesulfonates, revealed NrtT had an increase of 8 °C in its thermal stability in the presence of MOPS and almost 4 °C in the presence of HEPES and taurine (Fig. 4B,C). Circular dichroism analyses of NrtT in the absence and presence of taurine and MOPS still revealed the α–β profile characteristic of periplasmic binding proteins and structural changes in the presence of the ligands (Fig. 4D). Moreover, in the presence of taurine and MOPS, NrtT suffered quenching of tryptophans, suggesting they induce conformational changes in the protein and supporting the prediction that NrtT binds sulfonates (Fig. 4E). According to the structural model, the two tryptophans responsible for this behavior are externally positioned at domain I (W^72^) and domain II (W^134^; Fig. 4E, inset).

### Structural changes of NrtT in the presence of MOPS

**Figure 5:**
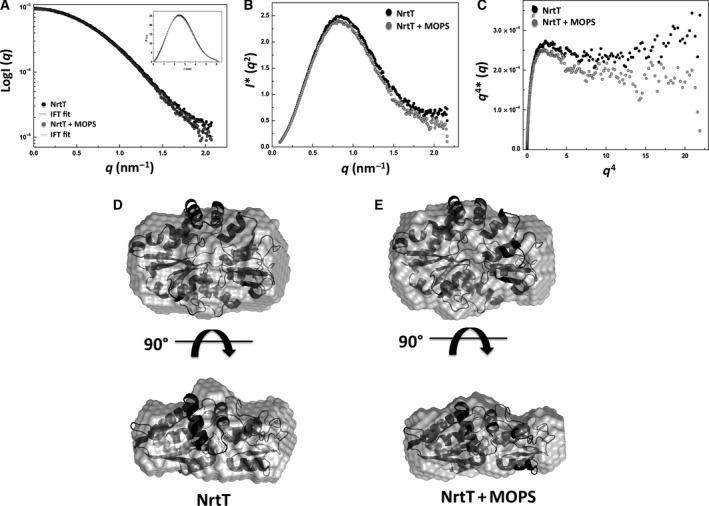
SAXS analysis of NrtT in the absence and presence of MOPS. (A) Experimental scattering curve and the best intensity fitting obtained from SAXS data. The inset shows the normalized pair‐distance distribution function *P*(*r*) showing a small difference between NrtT and NrtT + MOPS. (B) Kratky plot for the samples NrtT and NrtT + MOPS evidencing that in presence of MOPS NrtT is more globular. (C) Porod–Debye plot showing the interaction between NrtT and MOPS causes loss of flexibility. (D,E) *Ab initio* envelopes generated from the three‐dimensional structure model of NrtT and fitting of SAXS data by the rigid body optimization for NrtT (D) and NrtT + MOPS (E).

To monitor possible structural changes in NrtT in the presence of alkanesulfonate, SAXS analyses were performed for NrtT in the absence and presence of MOPS. Analysis from indirect Fourier transformation 35 and distribution distance function *P*(*r*) showed no changes in the conformation or oligomerization state of the protein in presence of MOPS (Fig. 5A). The gyration radius suffered slight alteration of 2.07–2.1 nm and the maximum distance changed from 6.1 to 6.3 nm. The scattering curves showed a slight difference between the samples in the presence and absence of MOPS in the high angles region, and according to the Kratky and Porod–Debye plots, in the presence of MOPS NrtT showed decreased flexibility and gain of folding (Fig. 5B,C). The fitting of SAXS data of NrtT in the absence and presence of MOPS with the molecular model of NrtT generated different envelopes that showed the protein suffers small structural changes and become more compact in the presence of the alkanesulfonate (Fig. 5D,E). Altogether, these data support the interaction of NrtT and MOPS.

### Deletion of the *nrtT* gene reduces gum production and pathogenicity to the citrus host

**Figure 6:**
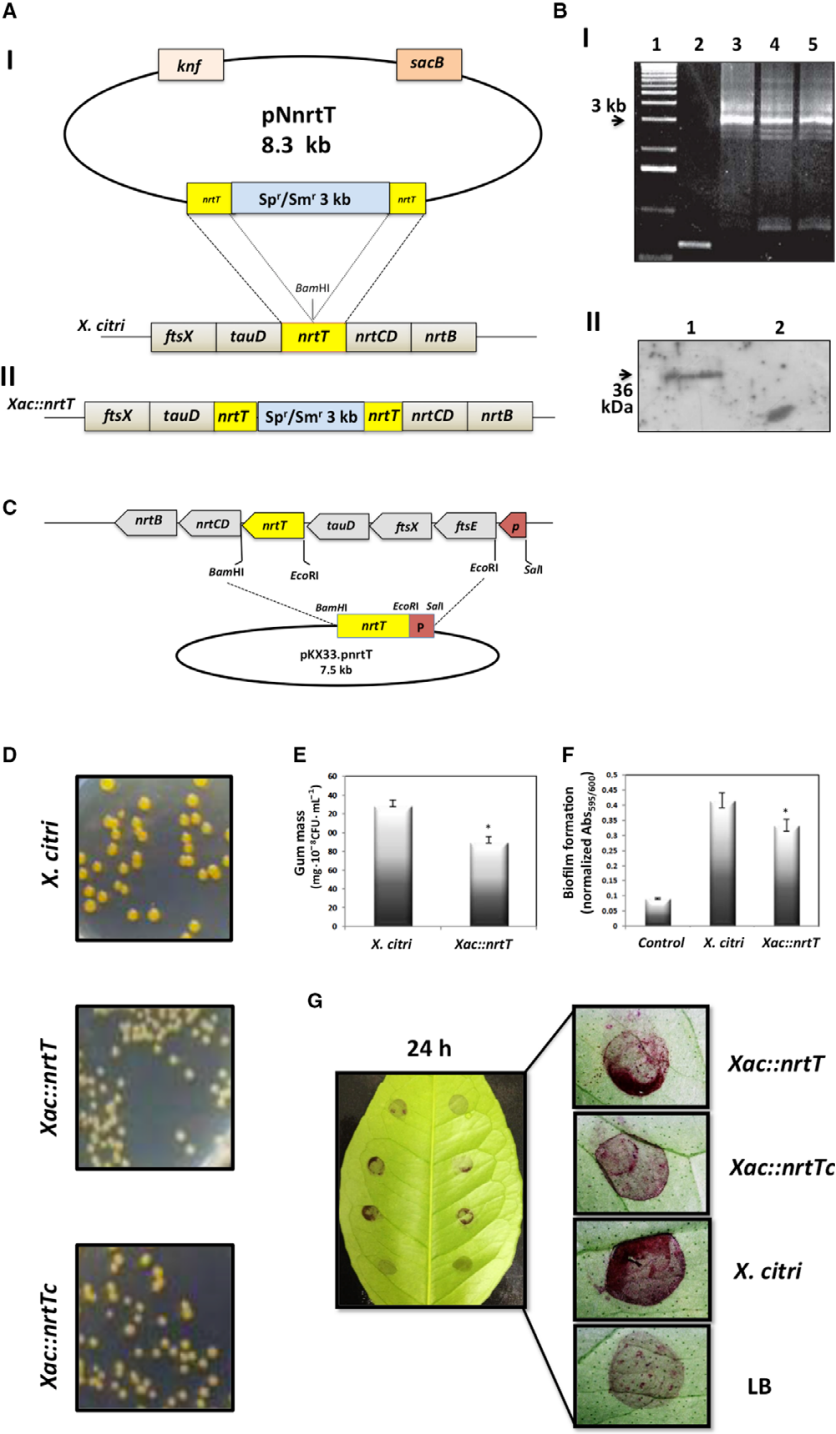
The role assessment of *nrtT* in the colonies’ phenotype, adhesion and biofilme formation. (A) Illustrative scheme of the construction of mutant (*Xac::nrtT*) and complementary (*Xac::nrtTc*) strains. (I) chromosomal deletion of the *nrtT* gene was obtained after electroporation of the suicide plasmid p.nrtT in *Xanthomonas citri* 306 strain and recombination for insertion at the *Bam* HI site of the *nrtT* gene of a 3 kbp fragment encoding spectinomycin and streptomycin resistances. (II) Final genome organization of the *Xac::nrtT* mutant. (B) Confimation of the mutant clones. (I) Products of the PCR reaction for amplification of the 3 kb Sp^r^/Sm^r^ cassette. 1, molecular mass marker; 2, PCR product using *X. citri* wild‐type DNA as template; 3–5, PCR product using putative mutant DNA as template, evidencing the amplification of a 3 kb fragment. (II) Western blot of the wild‐type and *Xac::nrtT* mutant 1 using the anti‐NrtT antibody. 1 and 2, total cellular extracts of *X. citri* and *Xac::nrtT* cells. (C) Scheme of the construction of the complementary strain *Xac::nrtTc*. (D) Morphology and color of the *X. citri* (Xac), *Xac::nrtT* and *Xac::nrtTc* colonies after growth in LB broth plates for 24 h. Colonies from mutant strains showed decreased size and attenuation of the color, which was partly reestablished by the complementary strain. (E) Xantham gum production by wild‐type and mutant strain in LB broth. A significant reduction of 26% was observed in the mutant strain (*P* < 0.05). (F) Biofilm production on abiotic surface (polypropylene plates) by *X. citri* and *Xac::nrtT* strains showing significant reduction in the mutant strain (*P* < 0.01). (G) Analysis of adhesion and biofilm formation on surface of *Citrus sinensis* leaves after 24 h of the cultures growth evidencing significant reduction in the mutant strain.

To evaluate the physiological role of NrtT *in vitro* and *in vivo*, we built an *X. citri nrtT*‐knockout (*Xac::nrtT*) using site‐specific inactivation of the *nrtT* gene with a suicide plasmid according to previously published procedures 12 (Fig. 6A,B). The complementary strain (*Xac::nrtTc*) was obtained after the transformation of pKX33 plasmid 36 carrying the *nrtT* gene under control of the promoter from the *X. citri tauDnrtTCDB* operon (Fig. 6C). The first evidence that *nrtT* deletion affected the phenotype of *X. citri* colonies was obtained in LB broth plates after 24 h growth. Colonies of the *Xac::nrtT* strain were smaller than the wild‐type and showed alteration in the color (Fig. 6D). This phenotype was previously observed in the *X. citri* mutant of the *ssuA* gene, which encodes the SsuA protein, and was related to a decrease in growth and xantham gum production 14. To test if *nrtT* deletion also affected the polysaccharide synthesis in the *Xac::nrtT* strain we measured the xantham gum produced by wild‐type and mutant strains after 24 h of growth in LB broth. *Xac::nrtT* showed a decreasing of 26% in xantham gum production in LB broth when compared with the wild‐type *X. citri* (Fig. 6E). Since xantham gum production is directly related to the ability of the bacterium to infect, colonize and form biofilm 37, 38, we analyzed the production of biofilm by *Xac::nrtT* in comparison with *X. citri* after growth of the cells in polypropylene plates and adhesion in sweet orange leaves (*C. sinensis*). The results showed a reduction of 24% in the values of absorbance indicating the *nrtT* deletion also affected biofilm formation (Fig. 6F). Similarly, the adhesion of *Xac::nrtT* in *C. sinensis* leaves after 24 h also showed reduction when compared with the wild‐type and recovery of the phenotype by the complementary strain was only partially obtained (Fig. 6G).

**Figure 7:**
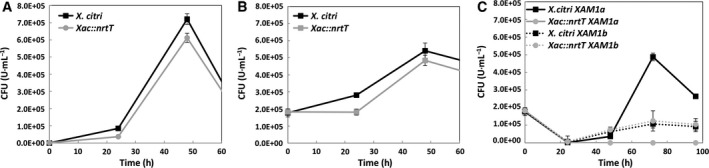
Role assessment of *nrtT* during *in vitro* growth. Growth curves of *Xanthomonas citri* and *Xac::nrtT* strains in (A) LB, (B) XAM1 and (C) XAM1 modified with 3.0 mm MOPS or 3.0 mm taurine replacing the ammonium sulfate to give XAM1a and XAM1b, respectively. Cells were growth at 30 °C, 250 r.p.m. for 72 h. The absorbance at 600 nm was measured every 24 h. The experiment was carried out in triplicate and the results represent the mean and standard deviation.

The effect of *nrtT* deletion in *X. citri* was monitored during *in vitro* growth of wild‐type and mutant strains in LB broth and XAM1 media. In both conditions, *X. citri* and *Xac::nrtT* strains showed a similar behavior, suggesting the *nrtT* gene is not essential for *in vitro* growth in these conditions where sulfur sources are not excluded or limiting (Fig. 7A,B). Based on the previous data that indicate MOPS and taurine as putative ligands of NrtT, *X. citri* and *Xac::nrtT* were grown in modified XAM1 medium, where the sulfur sources (originally 3 mm ammonium sulfate) were replaced, respectively, by 3 mm MOPS (XAM1a) and 3 mm taurine (XAM1b). The results showed that using taurine as sulfur source, both the wild‐type and the mutant strains had reduced but similar growth. On the other hand, *Xac::nrtT* was unable to grow when MOPS was used as a unique source of sulfur (Fig. 7C), indicating that NrtT is capable of taking up this alkanesulfonate.

**Figure 8:**
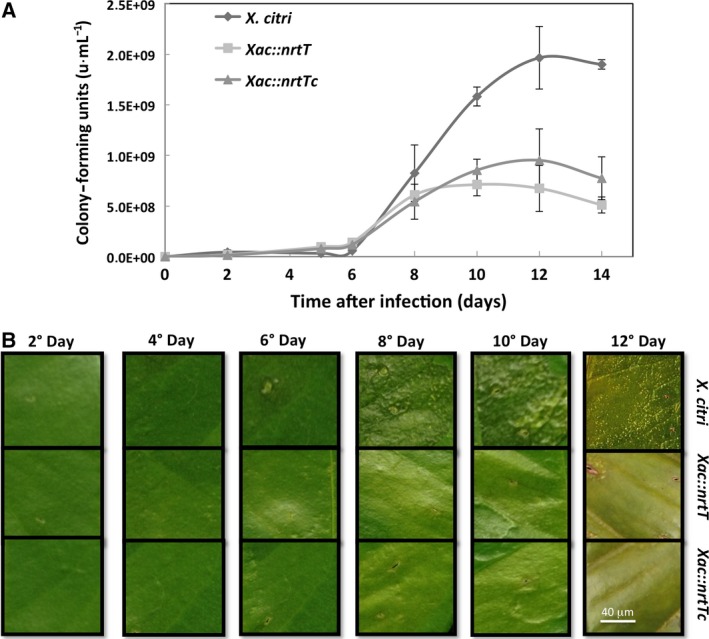
*In vivo* role assessment of *nrtT*. (A) Growth curves of the strains obtained after recovering the cells and plating in LB broth medium for 24 h. Sectors of a *Citrus sinensis* leaf inoculated with a syringe containing 100 μL of normalized CFU·mL ^−1^ of the strains *Xanthomonas citri*,* Xac::nrtT* and *Xac::nrtTc*. Three sectors from three distinct leaves were processed for each strain for replicates. (B) Development of the canker symptoms in *Citrus sinensis* leaves monitored during 12 days. With the lack of *nrtT* gene, *X. citri* mutant strain produced evident chlorosis and attenuated pustules when compared with the wild‐type strain. Complementation of the *nrtT* mutation with pKX33.pnrtT was not evidenced in these experiments corroborating the hypothesis of deletion of the full transporter by polar mutation. Leaves were photographed using a microscope at ×100 magnification.

To survey the effect of the *nrtT* deletion *in vivo*, we carried out inoculation of the three strains in *C. sinensis* leaves (orange plants) and monitored the growth and development of canker symptoms during 12 days. Cultures of the strains were inoculated in three sectors of the same leaves (Fig. 8A) and monitored during 12 days. *In vivo* growth was monitored from cells recovered from the sectors and plated in LB broth for 24 h. The deletion of the *nrtT* gene drastically affected the growth of the *Xac::nrtT* in *C. sinensis* plants in comparison with the *X. citri* and, again, the complementary strain was able to recover only part of the growth, which was still reduced (Fig. 8B). As a consequence of the decreased ability to grow of the mutant and complementary strains, the canker symptoms and phenotype evidenced by these strains after 12 days of inoculation were attenuated and evidenced only the chlorosis in comparison with the wild‐type (Fig. 8C), which besides chlorosis, induced formation of the typical pustules (Fig. 8C). These data are in accordance with the decrease of xantham gum, adhesion and biofilm formation, as previously shown.

## Discussion

In this work, we characterized the *X. citri* NrtT protein, a periplasmic component of the ABC transporter NrtCB putatively functioning in the transport of sulfonates, nitrate or taurine. In ABC transporter‐type importers, the periplasmic binding proteins are responsible for the affinity and specificity of the transport and define the function of the system. The bioinformatics analyses suggested NrtT was closely related to the five periplasmic binding proteins, from which three had alkanesulfonates as substrates. In fact, the superimposition of the structures showed the proteins shared a similar volume of the ligand pocket, discarding the bicarbonate and nitrate as putative ligands (see the pockets of CmpA and NrtA proteins). However, apart from Gly^51^, Gly^52^ and Gly^70^, which are conserved in the structures of *E. coli* and *X. citri*, the pocket does not show any other similarity. In *X. citri* SsuA, these residues are responsible for the coordination of the sulfate ion from the alkanesulfonates while the alkane chains are accommodated by interactions mediated by other residues from the pocket and water molecules 14. This observation suggests that NrtT might have different ligands when compared with SsuA. The presence of a gene encoding a taurine dioxygenase in the same operon reinforces this hypothesis and, in fact, *in vitro* assays using circular dichroism and fluorescence showed that only MOPS (an alkanesulfonate) and taurine (an aliphatic sulfonate) were able to increase the thermal stability and induce quenching of the tryptophans, strongly suggesting that the protein has some kind of interaction with these molecules. These data were supported by SAXS analyses, which also showed that the protein became more stable in the presence of MOPS. The envelopes generated from the apo and putatively bound proteins showed significant differences in volume and shape [as evidenced by changes in gyration radius, volume of particle and maximum diameter] indicating that there is a structural movement and packing of the protein in the presence of this ligand. In *E. coli* taurine is distinctively transported by the TauABC transporter, which also can be responsible for uptake of piperazine‐*N*,*N*′‐bis(2‐ethanesulfonic acid), 2‐(4‐pyridyl) ethanesulfonate, isethionate, 1,3‐dioxo‐2‐isoindolineethanesulfonate, 3‐aminopropanesulfonate, butanesulfonate and pentanesulfonate, but not MOPS or HEPES, which are substrates of the SsuABC transporter. In this bacterium, although propanesulfonate, hexanesulfonate and MOPS do not belong to the range of substrates of the TauABC transporter, TauD uses them as substrates for desulfonation 39. In accordance with this observation, the operon *tauDnrtTCDB* of *X. citri* encodes all the genes for transport and oxide‐reduction of the sulfonates. Altogether, the data obtained in this work suggest that *tauDnrtTCDB* is the third operon dedicated to uptake of sulfur compounds.

Using an *nrtT*‐knockout strain, we showed that NrtT plays an important role during *in vitro* and *in vivo* growth and that deletion of the *nrtT* gene provoked effects in *X. citri* similar to those observed in a strain carrying a deletion of the *ssuA* gene 14, such as small size and attenuated color of the colonies, deficiency of xantham gum production and adhesion, and development of attenuated symptoms of the disease in citrus leaves. The fact that the mutation affected xantham gum production is *per se* responsible for all the other effects. Production of xantham gum by the bacterium is an essential mechanism in the processes of infection and pathogenesis since this polysaccharide is responsible for attraction of liquids and nutrients, which favor colonization and biofilm formation 40. The deficiency in gum production affected the adhesion, colonization and pathogenesis as previously described for other *X. citri* mutants 41, 42. Although NrtT does not belong to the pathway for production of gum, all the enzymes belonging to it contain cysteines in their amino acid sequence. The deficiency in uptake of sulfur compounds would indirectly affect the available sources of this element for protein synthesis in general, and that would explain the slow bacterial growth in *C. sinensis* leaves. On the other hand, the inability of *Xac::nrtTc* to recover the wild‐type phenotype suggests some additional pleiotropic effect of the mutation, which is polar and may affect the genes downstream from *nrtT* producing a full‐transporter knockout or *ntrCDB* mutant. In addition, the stability of the plasmid *in vivo* and partial complementation of gene mutations in *X. citri* is often described in the literature 14, 43, 44, 45, 46. The fact that *Xac::nrtT* was unable to grow *in vitro* when MOPS was the unique source of sulfur strongly supports the effect of the gene, which also was corroborated by the biophysical analyses. Another key point to be detached is that we do not know what the real sulfonates present in the plants are and how the three different proteins (NrtT, SsuA1 and SsuA2) from *X. citri* deal with them *in vivo*. As for bacteria, sulfur is an essential element for plants, which rapidly convert the absorbed inorganic sulfate into organic molecules such as proteins, sulfonates and sulfolipids present in thylakoid membranes 47. In fact, the activation of the *sbpcysUWA* promoter that is responsible for sulfate uptake was drastically induced during infection and growth of *X. citri* in *C. sinensis* plants, evidencing lack of sulfate inside the leaves 18.

Although the precise role of alkanesulfonates during the growth of *X. citri* is not presently known, our results indicate that sulfur compounds, aliphatic sulfonates and alkanesulfonates have a previously unsuspected relevance for plant–bacterial interactions. It would be of interest to study how these systems evolved in this bacterium and the *Xanthomonas* genus and what their specific nutritional and physiological roles are.

## Author contributions

AB and AS designed the experiments; AS performed all the experiments with the exception of assays of adhesion and biofilm formation, which were performed by VRP, and mutant strain construction, which was performed by EEO; AB and AS wrote the manuscript and made revisions. AB supervised the manuscript.
